# Establishment of computational biology in Greece and Cyprus: Past, present, and future

**DOI:** 10.1371/journal.pcbi.1007532

**Published:** 2019-12-19

**Authors:** Anastasia Chasapi, Michalis Aivaliotis, Lefteris Angelis, Anastasios Chanalaris, Ioannis Iliopoulos, Ilias Kappas, Christos Karapiperis, Nikos C. Kyrpides, Evangelos Pafilis, Eleftherios Panteris, Pantelis Topalis, George Tsiamis, Ioannis S. Vizirianakis, Metaxia Vlassi, Vasilis J. Promponas, Christos A. Ouzounis

**Affiliations:** 1 Biological Computation & Process Lab, Chemical Process & Energy Resources Institute, Centre for Research & Technology Hellas, Thessalonica, Greece; 2 School of Medicine, Faculty of Health Sciences, Aristotle University of Thessaloniki, Thessalonica, Greece; 3 School of Informatics, Aristotle University of Thessaloniki, Thessalonica, Greece; 4 Botnar Research Centre, NDORMS, Medical Sciences Division, University of Oxford, Oxford, United Kingdom; 5 Division of Basic Sciences, School of Medicine, University of Crete, Heraklion, Greece; 6 School of Biology, Aristotle University of Thessaloniki, Thessalonica, Greece; 7 Department of Energy, Joint Genome Institute, Walnut Creek, California, United States of America; 8 Institute of Marine Biology Biotechnology and Aquaculture, Hellenic Centre for Marine Research, Heraklion, Greece; 9 First Psychiatric Clinic, Papageorgiou General Hospital, Aristotle University of Thessaloniki, Thessalonica, Greece; 10 Institute of Molecular Biology and Biotechnology, Foundation for Research and Technology Hellas, Heraklion, Greece; 11 Department of Environmental Engineering, School of Engineering, University of Patras, Patras, Greece; 12 Department of Pharmacology, School of Pharmacy, Aristotle University of Thessaloniki, Thessalonica, Greece; 13 Institute of Biosciences & Applications, National Centre for Scientific Research Demokritos, Athens, Greece; 14 Bioinformatics Research Laboratory, Department of Biological Sciences, University of Cyprus, Nicosia, Cyprus; University of Virginia, UNITED STATES

## Overview

We review the establishment of computational biology in Greece and Cyprus from its inception to date and issue recommendations for future development. We compare output to other countries of similar geography, economy, and size—based on publication counts recorded in the literature—and predict future growth based on those counts as well as national priority areas. Our analysis may be pertinent to wider national or regional communities with challenges and opportunities emerging from the rapid expansion of the field and related industries. Our recommendations suggest a 2-fold growth margin for the 2 countries, as a realistic expectation for further expansion of the field and the development of a credible roadmap of national priorities, both in terms of research and infrastructure funding.

## Introduction

In the mid-1980s, a generation of prominent biologists inspired some of their students to follow an interdisciplinary career in the life sciences. Back then, the technological advances in modern molecular biology represented the cutting edge, including DNA sequencing, RNA blotting, and protein purification. In addition, the faster and cheaper structure determination of biological macromolecules was emerging, first by X-ray crystallography and later by nuclear magnetic resonance. Another parallel world in the area of computation, statistical science, electrical engineering, and software development was also emerging across the 2 countries, with remarkable successes fueled by some of the best minds of the era.

Despite their most prudent guidance, our great teachers and dear professors did not—or, in fact, could not—easily anticipate the era when technology would start quantifying living matter through the use of laboratory instrumentation, microfluidics, robotic and computer systems as well as the corresponding algorithm implementations [1]. Some of us—by choice, accident, or both—picked a career path that was not exactly well-defined, at the intersection of the revolution of molecular biology and the explosion of information technology, in the late 1980s [2]. The nascent field did not possess a name yet, but there was a premonition that it would become a dominant force in the life sciences, known as computational biology [3].

We review the establishment of this field in Greece and Cyprus, over the past 40 years or so, in terms of research activities, education and training, service provision, and other community and infrastructure projects. We attempt to quantify and map national activity using the current bibliography and thus project it into the future. Finally, we issue a number of recommendations relevant for the 2 countries, taking into account the rich biodiversity and their particular geographical and geopolitical context, with the hope that policies can be formulated with more precision and insight for future development of bioinformatics in this southeastern, strategically located corner of Europe. We hope that this Perspective article will be used to record and amplify current efforts in the 2 countries, documenting the development of the field. Naturally, it is not feasible and certainly beyond the scope of this article to provide a detailed record of the activities that have contributed to a successful computational biology community in Greece and Cyprus; for this, we apologize and hope that additional efforts might be reported in the pertinent literature in the future.

## Background

Biological research in Greece inevitably followed the 2 main facets of the life sciences, namely, genetics—including zoology, botany, and ecology—and biochemistry—including molecular, cell, and structural biology. Within these activities, the development of computation in biology has emerged from 2 streams, first by structural biologists (e.g., Hamodrakas, Kokkinidis, Moudrianakis, and Tsernoglou) and second by evolutionary biologists, including figurehead scientists (e.g., Kafatos, Kastritsis, Rodakis, and Zouros). Similarly to Greece, and across Cyprus, a strong computer science and engineering community emerged, which provided a fertile ground for the—almost unforeseen—needs and requirements for computational biosciences in the recent past. Furthermore, with the introduction of high-throughput -omics technologies, more opportunities have arisen everywhere [4]. Greece and Cyprus, as developed economies, are now investing in -omics research and training, including genomics and metagenomics, transcriptomics and proteomics, systems and synthetic biology—all fueled and supported by fundamental research and technological applications in bioinformatics.

As reported in other cases—and in the context of this collection in *PLOS Computational Biology* [5–19], Brazil [20], India [21], and elsewhere [22–24], the role of smaller countries in the global scene takes a special dimension, creating both specific responsibilities and significant opportunities. A general conclusion from those reports is that small(er) countries need to support global initiatives, by contributing to materials and methods within international projects and, in return, obtain access to data and training resources as well as build their own national agendas for the use of new technologies in certain fields of regional priority.

### The early years: Establishment (1981 to 2000)

In the 1980s, some of us were fortunate enough to conduct our doctoral research in environments that were supporting biological computation and anticipated the modern era. There were real difficulties to identify a proper graduate program that had some elements of computation for biology, mostly biostatistics. In fact, the few graduate programs in biological computing of that era were oriented towards theoretical ecology or plant genetics and—less so—to molecular biology. A fertile interface between ecological genetics and molecular biology was phylogeny research, where some of us endeavored, inspired by such greats as Theodosius Dobzhansky and their influential successors. Shifting the development of technical skills in life science research exclusively to tools such as algorithms and databases was quite a risky career choice back then [25].

Even more so, focusing on subject matters of biological data analysis for computer scientists was an equally precarious move. Many of us can testify to certain memories of isolation and solitary confinement in various biology laboratories, fiddling with hardware and software of various kinds of that era. Funding opportunities were quite limited, with the exception of some elite institutions such as the European Molecular Biology Laboratory (EMBL), Human Frontier Science Program (HFSP), and others willing and able to invest in the development of computational biology through postdoctoral fellowship schemes and career-defining exchange visits that some of us benefited from during that early period.

With Greece being a member state of the EMBL, which supported the development of computational biology with its “BioComputing Programme” and later established the European Bioinformatics Institute (EBI; Cambridge, United Kingdom), more opportunities started to emerge. Incidentally, a “Training-and-Mobility-for-Researchers” (TMR) grant by the European Commission’s Framework Programme supported a number of laboratories and individuals who later became laboratory heads across Europe: Collaborative projects with many European laboratories and leading computational biologists were the result of this grant [26], with high-impact work published in the literature, receiving publicity from mass media, including newspapers and television in Greece. The term “bioinformatics” was becoming part of the common vocabulary, while the community was growing—reflected in an influential editorial for the wider public and the financial world [27]. A landmark event for Greece was the organization of the Intelligent Systems for Molecular Biology (ISMB) conference in 1997 in Chalkidiki, where the mission statement of the International Society for Computational Biology (ISCB) was formulated (see https://www.iscb.org/iscb-history). Incidentally, these local efforts would have faded away if the field had not experienced an explosion worldwide soon afterwards, at the turn of the 20th century.

### The next 10 years: Recognition (2001 to 2010)

By 2002 and with the support of senior leadership at the University of Crete, one of the first bioinformatics academic appointments in Greece was established. Around that time, a similar position was created at the University of Cyprus, heralding a new period with more support for the field through research and teaching programs at the undergraduate and graduate levels, later (2016) followed by a European Research Area (ERA)-Chair appointment at the Cyprus Institute of Neurology and Genetics. Aggregating around initiatives already formulated at the EMBL, such as EMBNet and the EBI (Research & Services) [28,29], including many training events for advanced graduates (bioinformatics workshop at Institute of Agrobiotechnology (INA)-CERTH 2006, ENFIN workshop 2007, and EMBO metagenomics workshop 2008), the field started receiving certain regional publicity and attracting the attention it deserved [30]. During this time, there was a general excitement worldwide, with investors willing to fund a range of start-up companies that became active in the fledgling industry [27].

The utter relevance to biomedical research and human health started emerging as a serious proposition, following the sequencing of the human genome [31]. In both countries, a number of medical departments started investing in the area, in collaboration with engineering and polytechnic schools—examples abound and include the universities of Athens, Crete, Cyprus, Ioannina, and Thessalonica. A key, exemplary collaboration was the involvement of the Institute of Molecular Biology & Biotechnology (IMBB) with the mosquito genome sequencing projects [32,33], which were followed up much later [34]. Other laboratories in this phase included the Computational Genomics Unit (probably the largest in Greece in 2006–2007) at the (now defunct) Institute of Agro-biotechnology at CERTH, with its roots at the EBI, among others [35].

Some of us can remember an informal gathering at ISMB 2004 in Glasgow, where colleagues from Cyprus and Greece came together to discuss a more coordinated effort to develop the field of computational biology in both countries—this resulted in the first, informal conference, HSCBB01, at Heraklion in 2006. The incorporation of a professional society called the Hellenic Society for Computational Biology & Bioinformatics (HSCBB; hscbb.gr) followed soon, and a series of conferences were organized subsequently with mixed success at times and an attendance of more than 100 participants at each event [36].

Towards the end of that decade and as the field was developing worldwide, Greece (2010)—followed by Cyprus (2012)—experienced an unprecedented economic crisis, out of which the former is still struggling to escape. There was already a widely held view that the field required infrastructure development far from the capacities of small countries, an insight that has led to arguments for larger communities and their active participation in relevant infrastructures and international consortia. A little earlier, the Mikrobiokosmos (MBK; mikrobiokosmos.gr) initiative was founded (2005), with the aim to decipher the microbial wealth of Greece’s natural habitats. Interaction of the MBK with the computational biology communities remains strong and is primarily supported by the University of Patras and the Hellenic Centre for Marine Research (HCMR) in Crete. Other interactions involved the Hellenic Crystallographic Association (HECRA), which co-organized training events on computational structural biology over the years—e.g., from the first congress of HECRA (2002) to a joint international conference of HECRA and the HSCBB (2016), among other activities.

### The past 10 years: Growth (2011 to 2020)

**Fig 1:**
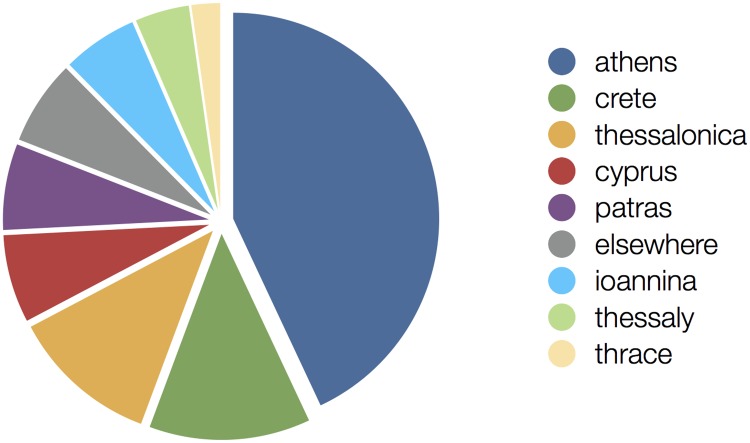
Bioinformatics activity in Greece and Cyprus. Relative distribution per region in Greece and Cyprus (entire country) in terms of volume of bioinformatics-related publications, as an indicator of research activity (measured until October 2018).

The interaction of multiple laboratories across Greece and Cyprus has helped us to develop the field, in collaboration with the state authorities. Various national, European, and international programs supported this development, probably in an ad hoc manner and not always with clear strategic planning. With laboratories in the main universities (following, indeed, their institutional names) at Athens, Crete, Thessalonica, Cyprus, Patras, Ioannina, Thessaly, Thrace, and elsewhere (Corfu, Chania, Chios, Kavala, Kozani, Serres, and Piraeus) (Fig 1), our community has emerged from the early, uncertain steps and acquired a respectable place in today’s landscape. For example, the HSCBB has co-organized during 2012 the Institute of Electrical and Electronics Engineers (IEEE) 12th International Conference on Bioinformatics and Bioengineering (BIBE2012) in Cyprus. In addition, strong support from our diaspora has been instrumental in forging international collaborations [37]. By then, the success of the field was already indisputable as part of the fabric of all life sciences [38].

In 2013, a new effort for a broader scientific society was instigated, augmenting the scope of the HSCBB, which we have called Hellenic Bioinformatics (HBio.info; http://hbio.info). For both societies, the name “Hellenic” signifies the adoption of a national, not state, community across the 2 countries and colleagues of the Greek Diaspora. HBio.info has organized more international conferences as follow-ups to the previous HSCBB efforts, with invited speakers from all over the world and the registration of approximately 300 participants at some of these conferences, so far. The newly established society was created with the aim of being more inclusive and expansive, opening the horizons to other regional groups in Southeastern Europe, the Middle East, and North Africa. The new HBio.info society has been supported by prominent colleagues abroad and became an Affiliate Group of the ISCB in 2017. Finally, the European Conference on Computational Biology (ECCB) 2018 took place in Athens, during September 2018, co-organized by the HSCBB, another landmark event for the community.

Training activities such as workshops and advanced courses by members of Hbio.info have been organized, co-organized or supported in Athens, Thessalonica, Izmir, and Tunis (funded by EMBO) [39], as well as Corsica (France), Beirut (Lebanon), and all the way to Mauritius (funded by H3AfricaBioNet) [40]. The EBI Roadshow took place in Nicosia (2009) as well as Athens and Thessalonica (2012), along with other EMBL-EBI off-site training activities, a valuable contribution. Another well-known workshop series on computational molecular evolution (https://cme.h-its.org/exelixis/teaching.html) has been supported by the HCMR where it took place every even year since 2010. In addition, undergraduate education curricula in most universities now include bioinformatics modules, while graduate programs relevant to the field exist at the universities of Athens, Crete, Cyprus, Thessalonica, Thessaly, and Patras. Participation in other research and training activities include the ISCB (as affiliate organizations), Goblet (global training infrastructure), Elixir-Europe (bioinformatics infrastructure via the Elixir-GR project), Helbionet (at HCMR), LifeWatch (HCMR), H3Africa (H3ABioNet, training) [40], Genomic Encyclopedia of Bacteria and Archaea (GEBA) [41], MetaSub (via CERTH) [42], and Euratom (jointly at AUTH & CERTH) [43]. Finally, editorial roles in the journals *Bioinformatics*, *PLOS Computational Biology*, *Microbial Genomics*, *Frontiers in Systems Microbiology*, and others have boosted our involvement in the field under various roles, as associate editors, reviewers and—of course—(co-)authors.

The status of Greece as an Observer State in the bioinformatics infrastructure European Strategy Forum on Research Infrastructures (ESFRI) project Elixir was triumphantly terminated in 2018, when—during the ECCB conference in Athens—it was announced that Greece was now a full member of this pan-European project, where the 2 Societies actively participate as Associated Organizations. Cyprus has also made a step towards membership, by becoming an Observer State in 2018, signifying the commitment of the country and its academia to join the international community in this European project. In fact, launching sustainable Elixir-related bioinformatics services might require new reward schemes, parallel to academic career paths [44].

## Recommendations and outlook

Given the aforementioned background and the significant efforts of the past and present, we can outline certain elements that pertain to small(er) countries and their role in this high-tech, big-science area that has transformed the life and medical sciences during the first decades of the 21st century [38]. In particular, as computational biology has permeated throughout all of biological, biomedical, and biotechnological research [38], requiring big data and large infrastructures both in terms of hardware, software, and ultimately people, we ask the question, “Where do our two countries stand in the global arena of this highly competitive area?” Quantifying output in terms of the number of publications as a simple productivity index, we envisage a future that our community faces with ambition and realism, not directly influenced by politics [45].

### The role of not-so-small countries

The field of bioinformatics has experienced an explosive growth in recent times mainly due to the expanding scope of genomic technology in its various incarnations. While these critical technologies have been developed in a handful of countries, computation with public-domain (and not bespoke and owned) biological data, with a lower-cost entry point, has propagated widely. Both these elements, the advancement of key technologies locally and the open-access data availability in international life science databases, present challenges and opportunities, not only for Cyprus or Greece but also other countries of similar size (in terms of population, gross domestic product (GDP), or GDP per capita—not shown) [46]. It is instructive, therefore, to assess the output of countries in terms of numbers of published papers so that realistic targets can be set for those lagging behind the “leaders.” While this facet should not be seen as a main objective of this Perspective article, a simple analysis reveals some interesting facts, which can be further refined in future work. The set goals, which can be a challenge even for the developed world, might also be used for strategies in less well-off countries with the support of local and international initiatives. Good examples include Brazil [6], China [10], Costa Rica [9], and Cuba [7]. Generally speaking, bioinformatics along the wider information technology industries can become a key transformative force for more traditional economies to engage in knowledge-based activities.

### Quantifying output and growth

To compare nations across the developed world, we have used cursory statistics from the Web of Science for 35 countries—namely the European Union 28 (EU-28) group [47], plus 7 countries where activities in the field are quite substantial: Australia, Canada, China, India, Japan, the United States, and Switzerland. We might have included New Zealand, Norway, Russia, or South Africa, but this would not affect the general pattern that emerges from this simple analysis—left for future work and a more global comparison. The keyword “bioinformatics” for all mentioned countries returns 37,388 publications, while the aforementioned group of 35 collectively returns 43,677 publications (joint publications are not excluded, therefore the higher count signifies a dense international collaborative network across those countries), thus covering pretty much the entire global output in the field. The US alone has produced more than 12,000 publications, one-third of the total. This impressive output needs to be normalized by econometric indices, such as population (in millions) and GDP (or GDP per capita—which, for those developed countries does not seriously affect the ranking. Then a slightly different, quite interesting picture starts emerging, with significant implications for future planning.

**Fig 2:**
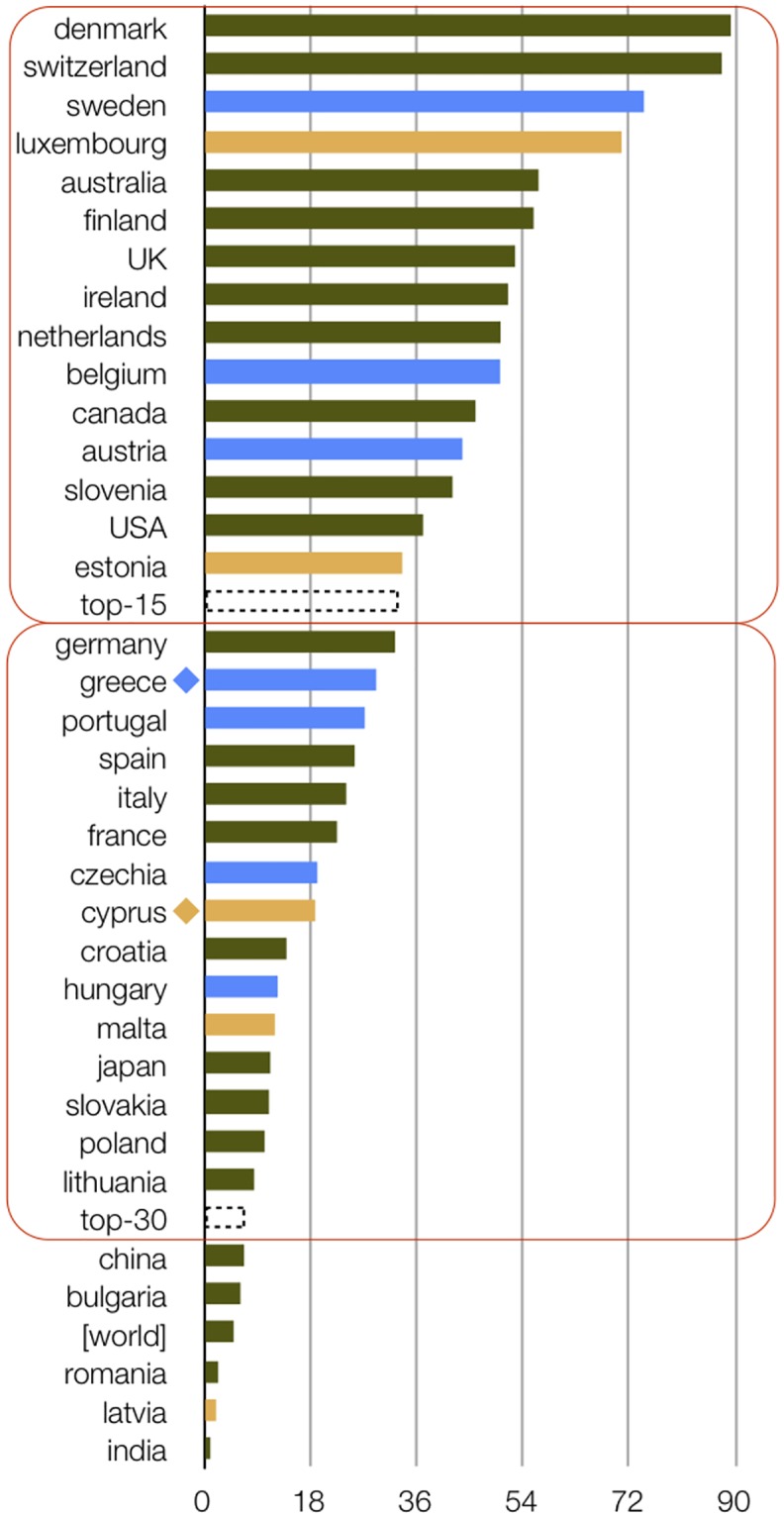
Output of publications per country (EU28 + 7 other countries), per million inhabitants. The 2 groups defined herein are marked by rectangles in a red outline. Greece is highlighted by a blue diamond and Cyprus by an orange diamond; countries with similar population to Greece (in blue) or Cyprus (in orange) are shown for comparison. Top-15 (>33 PpM), top-30 (>7 PpM), and world average (5 PpM, in square brackets) are also shown. EU28, European Union 28; PpM, publications per million.

When population is taken into account (Fig 2), there are 2 groups emerging as top 15 (more than 33 publications per million inhabitants [PpM]) and top 30 (rank positions 16–30; more than 7 PpM). In the leading group, we observe Denmark (89 PpM), followed by Switzerland (88 PpM), Sweden (75 PpM), Luxembourg (71 PpM, affected by a small population), Australia (57 PpM), Finland (56 PpM), and the UK (53 PpM), followed by Ireland (51 PpM), the Netherlands and Belgium (50 PpM), as well as Canada (46 PpM). The US (with the largest absolute output in the top group) is listed at 37 PpM (affected by a large population). The top 30 group includes Germany (32 PpM), Greece (29 PpM), Portugal (27 PpM), Spain (26 PpM), Italy (24 PpM), France (23 PpM), and Cyprus (19 PpM), among others. The world output is listed at 5 PpM, with China (7 PpM) and India (1 PpM) penalized for their large populations. While we do not argue that this is a complete analysis, this simple indicator suggests that leading nations with scores above 30 set the bar high for Greece (rank 17 out of 35) and Cyprus (rank 23 out of 35), which need to double their output in terms of number of publications as a realistic target. As this rarely happens within a stable community, the proposition is that the field has room for growth by 2-fold in our case, proportionally to leading nations. On the other hand, the PpM index comes as a pleasant surprise, suggesting that the contributions of our community to the global enterprise might be modest yet respectable—on a par with similar countries and economies and a proxy indicator of research and development (R&D) productivity that stimulates both innovation and learning [48].

### Institutions in Greece and Cyprus

We provide a reference to the academic institutions in Greece and Cyprus, as follows:

Greece: 49 academic institutions (49 public)
http://excellence.minedu.gov.gr/thales/en/institutions
For more information, see http://www.webometrics.info/en/Europe/greece.
Cyprus: 8 academic institutions (3 public, 5 private)
http://www.highereducation.ac.cy/en
For more information, see http://www.webometrics.info/en/Europe/cyprus.

These links are provided for completeness.

### Concluding remarks

As developed countries and economies, Cyprus and Greece need to compete and compare themselves with similar-scale counterparts, for example, Belgium, Austria, and Denmark (for Greece, in increasing order of GDP per capita) or Malta, Estonia, and Luxembourg (for Cyprus, likewise), taking into account geography and local context. Despite modest successes and certain false starts in the recent past, when the application of bioinformatics in domains of direct interest to society was not necessarily obvious, the landscape is now changing with the needs for large-scale analysis of big data for human health, biotechnology, bioengineering, and the environment—despite theoretical or practical issues that still linger [49]. Both countries enjoy a very high level of research, services, and education in life sciences, computing, medicine, and engineering, so that incorporation of novel technologies in the domains of -omics, synthetic biology, and translational medicine can directly benefit both research and applications in the near future. It is therefore logical to assume that investment in computational biology to support these critical areas in an interdisciplinary manner (cf. the UK experience [50]) will be of paramount importance for the well-being of both countries’ citizens and the creation of relevant industries with (investor-based) start-ups, (academia-based) spin-offs, and, perhaps, larger companies.

### Specific recommendations

Based on the aforementioned, and our collective experience over the years (e.g., [51]), we issue the following recommendations:

- Identify national priorities for future research (e.g., biomedicine, biodiversity)
- Expand research capacity of key laboratories, with documented, significant impact
- Support a small number of bioinformatics service components with global outreach
- Expand training at all levels, from undergraduate to graduate and advanced
- Connect with active industrial partners as stakeholders for future applications
- Enable a targeted effort for start-up companies jointly with venture capital
- Establish participation in selected international consortia and strategic actions

We hope that our recommendations will be useful for the design of national strategies in our countries and elsewhere.

### Future challenges

The potential for any “small” country to invest in computational biology is currently significant, especially in conjunction with the development of other technologies, such as biomedical or environmental genomics. Given the substantial presence of Greece and Cyprus in the fields of biomedicine and computing, it is crucial to commence a public dialogue for the further, coordinated development of bioinformatics to establish research, services, and training with notable contributions at the national, European, and international levels.
